# Candidiasis: Insights into Virulence Factors, Complement Evasion and Antifungal Drug Resistance

**DOI:** 10.3390/microorganisms13020272

**Published:** 2025-01-25

**Authors:** Nidaa Riaz Gaffar, Nisha Valand, Umakhanth Venkatraman Girija

**Affiliations:** Leicester School of Allied Health Sciences, Faculty of Health & Life Sciences, De Montfort University, Leicester LE1 9BH, UK

**Keywords:** *Candida* species, complement evasion, secreted proteases, complement regulators

## Abstract

Invasive fungal infections constitute a substantial global health burden, with invasive candidiasis representing approximately 70% of reported cases worldwide. The emergence of antifungal resistance among *Candida* species has further exacerbated this challenge to healthcare systems. Recent epidemiological studies have documented a concerning shift towards non-albicans *Candida* species, exhibiting reduced antifungal susceptibility, in invasive candidiasis cases. The complement system serves as a crucial first-line defence mechanism against *Candida* infections. These fungal pathogens can activate the complement cascade through three conventional pathways—classical, lectin, and alternative—in addition to activation through the coagulation system. While these pathways are initiated by distinct molecular triggers, they converge at C3 convertase formation, ultimately generating biologically active products and the membrane attack complex. *Candida* species have evolved sophisticated mechanisms to evade complement-mediated host defence, including the masking of cell wall components, proteolytic cleavage and inhibition of complement proteins, recruitment of complement regulators, and acquisition of host proteins. This review examines the intricate interplay between *Candida* species and the host complement system, with emphasis on complement evasion strategies. Furthermore, we highlight the importance of exploring the crosstalk between antifungal resistance and immune evasion strategies employed by *Candida* species. Understanding these interactions may facilitate the development of novel therapeutic approaches and strategies to overcome treatment failures in *Candida* species infections.

## 1. Introduction

Fungal pathogens pose a substantial burden on human health. Invasive fungal infections have emerged as a significant and escalating global health concern, largely attributed to the increasing prevalence of susceptible populations [1]. Global estimates reveal that an estimated 1.9 million individuals are affected by acute invasive fungal infections annually, while an additional 3 million suffer from chronic severe fungal infections [2,3]. The growing incidence of antifungal-resistant species together with fungal co-infection with other pathogens further compounds this public health challenge. Candidiasis is a healthcare-associated opportunistic infection caused by the fungi of the genus *Candida*, which are typically commensal organisms found in the human gastrointestinal tract, oral cavity, female genital tract, and skin [4]. In Europe, cases of candidiasis and aspergillosis predominate among invasive fungal infections, with invasive candidiasis alone accounting for nearly 70% of all cases globally [3,5]. The COVID-19 pandemic has highlighted invasive fungal infections as a critical complication in severely ill, hospitalised patients. The three primary groups of fungal pathogens associated with COVID-19 co-infections are *Aspergillus*, *Mucorales*, and *Candida* species. *Candida albicans* as well as emerging pathogen *Candida auris* has been commonly implicated in COVID-19 co-infections [6,7].

The six most clinically relevant *Candida* species include *C. albicans*, *Candida parapsilosis*, *Candida krusei* (currently named *Pichia kudriavzevii*), *Candida tropicalis*, *C. auris*, and *Candida glabrata* (currently named *Nakaseomyces glabratus*) [8]. It is important to note that *C. krusei* and *C. glabrata*, while commonly grouped with classic *Candida* species, belong to distinct phylogenetic classifications. In 2022, the World Health Organisation (WHO) released a list of fungal priority pathogens, categorising them into three priority groups. In this classification, *C. auris* and *C. albicans* are designated as critical pathogens. *C. glabrata*, *C. parapsilosis*, and *C. tropicalis* are classified into the high-priority group, and *C. krusei* is placed in the medium-priority group [9].

Infections occur when these organisms breach the skin or mucosal barriers, often following iatrogenic procedures or in the presence of underlying medical conditions [7,8]. Candidiasis manifests in different forms: superficial infections like oral and vulvovaginal candidiasis or more severe, systemic infections classified as invasive candidiasis [7,10]. Interestingly, elevated *Candida* colonisation has been documented in various autoimmune conditions, including inflammatory bowel disease and psoriasis [11,12].

Although *C. albicans* remains the most common causative agent of invasive candidiasis, recent epidemiological trends indicate a significant increase in the incidence of invasive candidiasis caused by non-albicans *Candida* species. This shift in epidemiology can be attributed to the reduced susceptibility exhibited by these emerging pathogens to conventional antifungal agents [13,14]. Currently available antifungal treatment options are limited to three main classes—azoles, polyenes, and echinocandins—each with distinct mechanisms of action [15]. The emergence of multidrug-resistant species, such as *C. auris* and *C. glabrata*, poses a significant therapeutic challenge, potentially eliminating treatment options entirely. This not only complicates patient care but also enhances the ability of resistant strains to persist in healthcare environments. These emerging resistance patterns underscore the need for effective management strategies and antifungal stewardship programmes to combat drug resistance and preserve the efficacy of existing antifungal agents. Furthermore, the available antifungal treatment options are associated with significant host toxicity. These drugs can adversely affect multiple organ systems, particularly the nervous system, gastrointestinal system, and urinary system [16].

In response to *Candida* species invasion, the host mounts a multifaceted immune response, encompassing components of both innate and adaptive immunity. The innate immune response, characterised by the activation of dendritic cells, neutrophils, and macrophages, as well as the activation of all three pathways of complement, represents the first line of defence [17]. The complement system comprises a complex network of approximately 40 proteins [18]. The majority of these are synthesised primarily in the liver, with additional production by macrophages and other cell types [19]. These complement proteins recognise invading *Candida* species through pathogen-associated molecular patterns and facilitate host defence through multiple mechanisms: the opsonisation of microbes, the promotion of phagocyte recruitment, direct microbial lysis, and the initiation of the adaptive immune response. Notably, the complement system exhibits variable recognition and response patterns across different *Candida* species, which may contribute to their differential pathogenicity. The adaptive immune response, characterised by both cell-mediated and humoral responses, provides memory-based protection against recurrent infection [20].

Despite these robust host defences, certain *Candida* strains have evolved mechanisms to evade host defences and establish persistent infections. This review focuses on the interplay between *Candida* species and the complement system, a crucial component of innate immunity, and elucidates the strategies employed by *Candida* species to evade complement-mediated clearance. By delineating the complement evasion strategies utilised by *Candida* species, this review aims to enhance our understanding of fungal pathogenesis and the implication of these evasion mechanisms on current antifungal therapies.

## 2. The Arsenal of *Candida* Virulence Factors

*Candida* species exhibit a diverse array of virulence factors that enable them to establish infections and evade host immune responses. While *C. albicans* has been extensively studied and its virulence factors well characterised, several studies have also been conducted to elucidate the virulence mechanisms of non-albicans *Candida*. Across the *Candida* genus, common virulence factors include morphological changes, biofilm formation, and the production of hydrolytic enzymes [21]. Table 1 summarises the virulence factors among different *Candida* species.

### 2.1. Morphological Transitions

Morphological plasticity is a critical virulence mechanism among *Candida* species, enabling adaptation to diverse host environments. *C. albicans* possesses the ability to transition between yeast, hyphal, and pseudohyphal forms—a process integral to its pathogenesis, with the hyphal form being particularly detrimental to the host. Additionally, *C. albicans* undergoes white-to-opaque switching, a process which involves a heritable and reversible transition between white, oval-shaped cells and elongated, grey cells [17]. The virulence of white and opaque cells varies depending on the site of infection. For instance, white cells are more virulent in disseminated infections, while opaque cells are more virulent in cutaneous infections. Furthermore, opaque cells are less efficiently phagocytosed by immune cells compared to white cells. This suggests that the switching mechanism is not only a strategy to optimise pathogenicity but also a means to enable the evasion of host defences, based on the host’s physiological conditions [22].

Phenotypic plasticity is not unique to *C. albicans*—white-to-opaque switching is also observed in *C. tropicalis* [23]. However, morphological diversity varies among *Candida* species. *C. parapsilosis* exhibits a more limited range of morphological forms, only existing as yeast or pseudohyphae cell morphologies [24]. In contrast, *C. auris* does not produce hyphae or pseudohyphae under normal conditions; however, under high salt conditions, it can form basic pseudohyphae [25]. Studies have demonstrated that these morphological variants elicit distinct immune responses, with certain forms exhibiting enhanced resistance to the immune system [26]. Notably, *C. auris* infection results in higher fungal loads and persistent colonies compared to *C. albicans* [27]. Furthermore, studies have shown that neutrophils have a reduced ability to phagocytose and kill *C. auris* compared to *C. albicans*, due to the protective properties of its outer cell wall mannan layer [28,29,30]. Unlike most *Candida* species, *C. glabrata* does not exhibit hyphal growth, which distinguishes its virulence mechanisms from those of other members of the genus [31]. Despite its lack of hyphal growth, *C. glabrata* compensates through intracellular persistence within macrophages, differing from *C. albicans* that actively escape macrophages through hyphal growth and phagocyte piercing [32].

### 2.2. Biofilm Formation

Biofilm formation is a hallmark virulence factor of *Candida* species because of its ability to provide a protective matrix that shields the *Candida* cells from the host immune system and limits the penetration of antifungal agents [33]. The presence of biofilms significantly impairs the phagocytic activity of neutrophils and supresses their capacity to initiate downstream immune responses, further contributing to fungal persistence. *C. albicans* forms robust biofilms composed of yeast, pseudohyphal, and hyphal cells, which enhance its persistence and resistance [34]. Interestingly, recent studies have demonstrated that *C. tropicalis* exhibits a higher biofilm formation capacity compared to *C. albicans* [35]. In contrast, *C. auris* exhibits reduced biofilm formation efficiency relative to *C. albicans* [25]. *C. parapsilosis* also forms biofilms; however, its biofilm morphology differs from other *Candida* species due to the absence of true hyphal formation. Consequently, this structural variation results in a reduced biofilm volume compared to other *Candida* species [36]. Similarly, while *C. glabrata* forms biofilms, it lacks the hyphal structures characteristic of *C. albicans* biofilms [37]. Interestingly, *Candida* species biofilms exhibit upregulated efflux pump expression, which contributes to virulence by preventing the intracellular accumulation of toxic substances including heavy metals and reactive oxygen species, thereby enhancing their survival [35].

**Table 1:** Comparison of unique characteristics and key virulence factors among different *Candida* species.

Species	Unique Characteristics	Key Virulence Factors	References
*C. albicans*	White cells more virulent in disseminated infections; Opaque cells more virulent in cutaneous infections	Yeast–hyphal transition White-to-opaque switching Adhesins (Als3, Hwp1) Saps (1–10) Candidalysin Biofilm formation	[17,22,38]
*C. tropicalis*	Higher biofilm formation capacity than *C. albicans*; More potent candidalysin than *C. albicans*	White-to-opaque switching Adhesins Sapts (1–4) Candidalysin Biofilm formation	[23,35]
*C. auris*	Exists in either filamentous, pseudohyphal, or yeast cell morphologies; Decreased phagocytosis by neutrophils compared to *C. albicans*	Adhesins Sapas (1–7) Biofilm formation Thermotolerance Salt tolerance	[25,26,28,29,30,39]
*C. parapsilosis*	Exists in either yeast or pseudohyphae cell morphologies	Sapps (1–3) Lipases (Lip1 and Lip2) Biofilm formation	[24,31,36]
*C. glabrata*	Persistence and survival inside macrophages; No hyphal growth	Yapsins (1–11) Biofilm formation	[31,32,40]

### 2.3. Aspartyl Proteases and Other Hydrolytic Enzymes

The production of hydrolytic enzymes, encompassing secreted aspartyl proteases (Saps), phospholipases, hemolysins, coagulases, esterases, and lipases, is another key virulence factor in *Candida* species. Among these, phospholipases, particularly B1 and B2, play a crucial role in host cell destruction [35]. Notably, phospholipase activity is significantly higher among *Candida* species that produce biofilms [13]. Additionally, hemolysins enhance virulence by facilitating iron acquisition, which is essential for pathogen metabolism, growth, and persistence.

*C. albicans* produces adhesins such as the agglutinin-like sequence protein (Als3) and hyphal cell wall protein (Hwp1), which facilitate its adherence to host tissue. Notably, *C. tropicalis* upregulates the production of Als3 and Sapt1, demonstrating a strong adhesion capacity that sometimes surpasses that of *C. albicans*. The secretion of Saps (encoded by 10 *SAP* genes) and the production of the hyphal-specific toxin, candidalysin, further contribute to *C. albicans* virulence. While *C. tropicalis* produces Sapt1–4 as well as candidalysin, which is more potent than that of *C. albicans*, it remains less pathogenic overall. This can be attributed to several factors such as gene expression levels, pathogenic mechanisms, host interactions, and strain variability [35,38].

In contrast to *C. albicans* and *C. tropicalis*, *C. auris* lacks candidalysin and Hwp1 but possesses 14 proteins containing the aspartic peptidase domain. Among these, seven proteins (Sapa1–7) were selected for detailed analysis, and Sapa3 was identified as a major aspartyl proteinase involved in this species’ virulence [39]. *C. auris* demonstrates increased survivability at higher temperatures, correlating with increased aspartyl protease secretion [25]. *C. parapsilosis* potentially has 14 aspartic-protease-encoding sequences, although only the genes of *SAPP1–3* have been confirmed. It also produces lipases (Lip1 and Lip2) that enhance its pathogenicity [31,36]. In contrast to other *Candida* species, *C. glabrata* does not secrete proteases like Saps, but expresses cell wall-associated aspartic proteases known as yapsins, with at least 11 yapsin-encoding genes identified. These yapsins play multiple roles in *C. glabrata* virulence by maintaining cell wall integrity, regulating adhesin levels for host tissue attachment, controlling protein secretion, and ensuring the proper trafficking of vacuolar hydrolases [40]. Importantly, Saps contribute significantly to fungal pathogenicity by modulating host immune responses, particularly through their ability to interfere with complement proteins (see Section 3.3).

While *Candida* species employ various virulence factors to establish and maintain infection, the host immune system, including the complement system, plays a crucial role in defending against these opportunistic pathogens.

## 3. Complement Cascade and *Candida*: From Activation to Evasion

*Candida* and other fungal species can initiate complement activation through three well-characterised pathways: the classical, alternative, and lectin pathways. Although each pathway is initiated by distinct molecular triggers, they converge at C3 convertase formation, a pivotal enzyme in the complement cascade. The activation of these pathways results in the generation of biologically active products, including anaphylatoxins (C4a, C3a, and C5a) that orchestrate inflammatory responses and the membrane attack complex (MAC) that mediates the direct lysis of target cells [17,41,42]. Complement deficiencies, such as C3 and C5a deficiencies, are correlated with increased susceptibility to candidiasis, underscoring the complement system’s essential role in defending against both superficial and systemic *Candida* infections [43,44].

### 3.1. Multiple Routes of Complement Activation by Candida

The classical pathway initiates when C1q, a component of the C1 complex, recognises antigen–antibody complexes on microbial surfaces by binding to the Fc portion of immunoglobulins. Anti-mannan IgG and IgM antibodies, prevalent in the serum of most adults, are particularly crucial in activating this pathway against *Candida* species [17,45]. This interaction triggers the activation of C1r and C1s serine proteases, thereby initiating the complement cascade. Moreover, pentraxins—a family of pattern recognition molecules, including the serum amyloid P component—can bind *C. albicans* surfaces and activate the classical pathway through C1q interaction, providing an antibody-independent mechanism of activation [46].

The lectin pathway is activated upon the recognition of specific pathogen surface structures. The *Candida* cell wall, comprising an inner layer of chitin and glucans (β-1,3 and β-1,6) and an outer layer of mannoproteins [47], is integral to the immune response, facilitating interactions with host cells and subsequent immune activation. Mannose-binding lectin (MBL), complexed with MBL-associated serine proteases (MASP-1 and MASP-2), recognises terminal mannose residues, L-fucose, and N-acetylglucosamine (GlcNAc) on pathogen surfaces. Collectin-11, a C-type lectin that possesses collagen-like and carbohydrate recognition domains, similarly engages with pathogen surfaces through L-fucose and mannose residues, among other possible ligands [48,49]. Ficolins, structurally similar to MBL, also participate in this pathway by recognising GlcNAc and lipoteichoic acid (LTA). Various *Candida* species exhibit strong MBL binding, effectively triggering the lectin pathway [50]. Upon recognition, MASP activation initiates downstream proteolytic events that mirror those of the classical pathway [51]. Additionally, surfactant protein D (SP-D), a C-type lectin sharing structural and functional similarities with complement proteins, binds and agglutinates *Candida* species, enhancing phagocytic clearance without direct complement activation [52].

Unlike its counterparts, the alternative pathway has the capacity of self-initiation. This pathway is triggered when C3 is hydrolysed to C3b in the presence of factor B, factor D, and properdin, which then binds to hydroxyl and amine groups on microbial surfaces [53]. During *Candida* infections, immune-cell-mediated cell wall damage exposes inner glucans, promoting C3 accumulation and establishing glucan as a natural activator of the alternative pathway [54].

**Figure 1:**
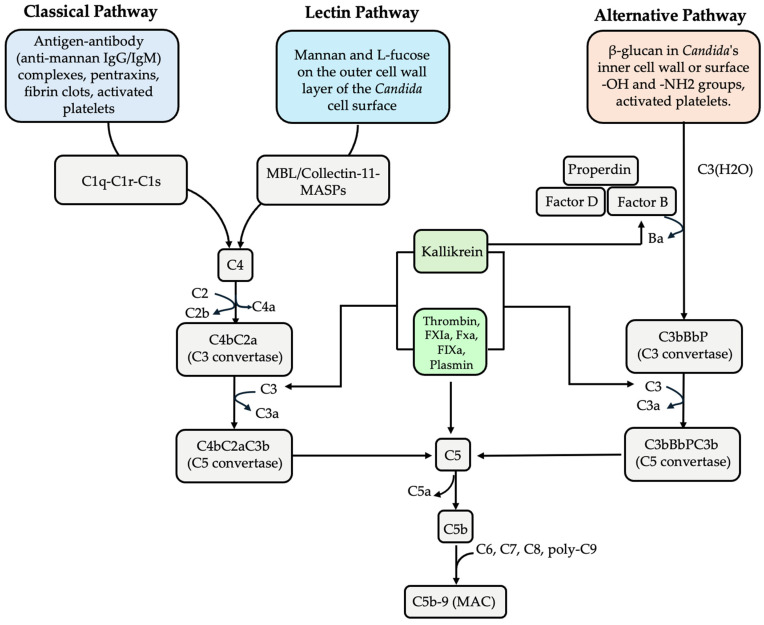
Overview of complement activation pathways and their interactions with the contact, coagulation, and fibrinolytic systems. This figure illustrates how *Candida* activates the three main complement pathways: classical (left), lectin (middle), and alternative (right). During activation, proteolytic cleavage generates anaphylatoxins (C4a, C3a, and C5a). All three pathways converge at C3 convertase formation, leading to C5 convertase assembly. This results in the generation of the membrane attack complex (MAC). The figure further illustrates how components of the contact system (kallikrein), coagulation system (thrombin, FXIa, FXa, and FIXa), and fibrinolytic system (plasmin) can directly cleave C3 and C5, providing alternative routes of complement activation. Additionally, kallikrein can cleave factor B, initiating the alternative pathway.

Beyond these traditional pathways, *Candida* species activate complement through indirect mechanisms involving the contact, coagulation, and fibrinolytic systems [55]. It has been demonstrated that activated platelets trigger both classical and alternative pathways, while thrombin directly activates C3 and C5 independently of conventional pathways. Additionally, fibrin clots also activate the classical pathway [56]. In the contact system, activated factor XII (FXIIa), generated by *Candida* surface contact or *Candida*–derived proteases such as *C. albicans* Sap2 and *C. parapsilosis* Sapp1, catalyses the conversion of prekallikrein, a precursor molecule, to kallikrein, a trypsin-like protease [57]. Kallikrein cleaves C3, generating its biologically active fragments, which contribute to C3 convertase formation. Moreover, kallikrein cleaves factor B, yielding Bb and Ba fragments which initiate the alternative pathway [58]. Within the coagulation cascade, FXIIa-driven thrombin generation enables direct C5 cleavage, forming functional C5 convertase independently of C3 [59]. Factors XIa, Xa, and IXa also effectively cleave C3 and C5 [60]. In the fibrinolytic system, *Candida* species utilise the surface proteins phosphoglycerate mutase 1 (Gpm1) and pH-regulated antigen 1 (Pra1) to bind plasminogen, facilitating its conversion to active plasmin [55]. Plasmin subsequently activates complement through C3 and C5 proteolysis [60]. Notably, some studies indicate that plasminogen may function as a complement inhibitor (see Section 4.2), suggesting a context-dependent role for plasminogen in complement regulation [61]. Figure 1 illustrates the activation mechanisms for all three pathways as well as their interactions with the contact, coagulation, and fibrinolytic systems.

*Candida* species have evolved sophisticated strategies to evade complement activation and facilitate their dissemination within the host. Primary evasion strategies employed by *Candida* species include the masking of cell wall components as well as cleaving and blocking complement proteins (Table 2).

### 3.2. Masking of Candida Cell Wall Components to Escape Complement

Intact *C. albicans* yeast cells exhibit intrinsic resistance to the alternative pathway of complement activation, which is attributed to the mannan-enriched outer layer of the cell wall masking the β-glucans in the inner layer. This immune evasion strategy is particularly significant in individuals with MBL deficiency, as their lectin pathway of complement activation is also compromised. MBL-deficient mice have been shown to be highly susceptible to *Candida* infections [62,63]. Several studies in humans have shown a correlation between MBL concentrations and recurrent *Candida* infections, particularly vulvovaginal candidiasis and peritonitis [64,65]. These emphasise the crucial role of the MBL-mediated lectin pathway of complement in eliminating *Candida* cells. Interestingly, this resistance can be overcome by the presence of anti-mannan antibodies, which activate the alternative pathway of complement by accelerating the deposition of C3b specifically on the antigen-binding fragments (Fab) of antibodies, thereby stabilising C3 convertase [45].

It is noteworthy that the degree of masking varies among *Candida* species. For instance, *C. glabrata* exhibits more pronounced masking compared to *C. albicans* due to a denser outer layer of mannoproteins in its cell wall [66]. Similarly, the cell wall of *C. auris* is characterised by a higher mannan density and low β-glucan proportions relative to *C. albicans*. Consequently, this composition may result in heightened lectin pathway activation while simultaneously inhibiting alternative pathway activation through β-glucan masking [67]. In contrast, *C. parapsilosis* exhibits a lower mannan content and increased β-glucan exposure. This structural characteristic suggests that β-glucan is more accessible in *C. parapsilosis* compared to in *C. albicans*, potentially enhancing its ability to activate the alternative pathway [68].

### 3.3. Cleaving and Blocking Complement Proteins by Candida Proteases

Another evasion mechanism employed by certain *Candida* species involves the cleavage and inhibition of complement proteins through the action of specific fungal proteases. As mentioned above, one of the key virulence factors of *C. albicans* is its capacity to produce Saps. Notably, Sap1, Sap2, and Sap3 have been demonstrated to degrade host complement proteins C3b, C4b, and C5, and inhibit the formation of MAC, thus blocking the classical and alternative pathways of complement [69]. Additionally, Sap2 is also known to cleave most immunoglobulins including IgG, which is important for complement activation [70]. Sap2 also exhibits proteolytic activity against IgA, an essential immunoglobin in mucosal immunity, thereby potentially compromising host defence at mucosal surfaces [71]. Furthermore, specific sequence variations in *C. albicans* Sap2, particularly the substitution V273L, are associated with enhanced pathogenicity. This increased virulence is attributed to the increased degradation of C3 and C3b, resulting in reduced complement activation [72].

The Sap family’s role in complement evasion extends to other *Candida* species as well. While Saps share sequence homology across different *Candida* species, they differ in their expression patterns and functional properties. Studies have demonstrated that Sapt1 from *C. tropicalis* effectively cleaves MBL and collectin-11, thereby preventing the activation of the lectin pathway of complement. Additionally, Sapt1 has been shown to cleave DC-SIGN (Dendritic Cell-Specific Intercellular Adhesion Molecule-3-Grabbing Non-integrin), a lectin receptor present on dendritic cells that recognises mannan residues. This cleavage impairs the immune system’s ability to detect and eliminate the fungal pathogen [73]. Similarly, Sapp1 and Sapp2 produced by *C. parapsilosis* effectively cleave and degrade C3b and C4b, thereby preventing the formation of C5 convertases. Interestingly, Sapp3 does not possess the ability to cleave complement proteins [74].

In addition to Saps, *Candida* Pra1, a surface-bound and secretory protein, is also involved in complement evasion. In *C. albicans*, Pra1 binds to and cleaves C3 at a site that is distinct from the target site of C3 convertase, generating C3a-like (C3aL) and C3b-like (C3bL) proteins. C3bL is degraded by complement regulators, namely, factor I (FI) in the presence of factor H (FH), preventing it from effectively participating in opsonisation, while C3aL lacks the necessary C-terminal arginine for receptor binding, effectively blocking downstream signalling pathways. This disruption of C3 function impairs the immune response, allowing *C. albicans* to survive and proliferate within the host [75]. *C. tropicalis* Pra1 (CtPra1), like its *C. albicans* counterpart, binds to complement proteins C3 and C3b, thereby inhibiting complement activation [76]. However, further studies are needed to determine whether CtPra1 cleaves C3 or C3b.

**Table 2:** Primary mechanisms by which *Candida* species evade complement system.

Complement Evasion Strategy	Mechanism	Species	References
Masking of cell wall Components	Outer mannan layer conceals β-glucans, reducing exposure to immune cells and complement system	*C. albicans* *C. glabrata* *C. auris* *C. parapsilosis*	[45] [66] [67] [68]
Cleavage and/or inhibition of complement proteins	Secreted aspartyl proteases (Saps) degrade complement proteins - Sap1, Sap2, and Sap3 degrade C3b, C4b, and C5 - Sap2 cleaves IgG and IgA - Sapt1 cleaves MBL and collectin-11 - Sapp1 and Sapp2 degrade C3b and C4b	*C. albicans* *C. albicans* *C. tropicalis* *C. parapsilosis*	[69] [70] [73] [74]
	Pra1 cleaves C3, preventing C3b formation and generating non-functional C3a-like peptides	*C. albicams*	[75]
	Pra1 binds C3 and C3b	*C. tropicalis*	[76]

## 4. Complement Cascade and *Candida*: From Regulation to Evasion

Maintaining homeostasis and health requires a delicate balance between complement activation and inhibition. The dysregulation of this balance has been implicated in various pathological conditions, including autoimmune disease and inflammatory disorders [53].

The complement system is tightly regulated by a network of plasma-soluble and membrane-bound regulators. Complement regulatory proteins act at various stages of the complement cascade through distinct biochemical mechanisms [77,78]. Complement regulation primarily occurs through two mechanisms: decay acceleration, which involves the dissociation of complement convertase complexes, including C3 and C5 convertases, and cofactor activity, which refers to the enzymatic inactivation of C4b or C3b, with the help of various cofactor molecules such as membrane cofactor protein (MCP), complement receptor 1 (CR1), and factor H (FH) [79].

### 4.1. Human Complement Regulators—Mechanisms of Action

The C1 inhibitor, a plasma protein, regulates the classical and lectin pathways by inactivating C1r, C1s, MASP-1, and MASP-2. The C4-binding protein (C4BP), another key fluid-phase regulator of these pathways, targets C4b and facilitates its cleavage into C4c and C4d. The resulting C4d fragment limits the amplification of complement activation, thereby contributing to the regulation of these pathways [80]. Factors H and I are key plasma regulators of the alternative pathway [46]. FH, composed of 20 short consensus repeats (SCRs), each with distinct functions, exhibits decay-accelerating activity. SCRs 1–4 bind C3b and possess decay-accelerating activity, while SCRs 6–8, 19, and 20 bind to host surfaces [81]. FH acts as a cofactor for FI in C3b inactivation and regulates C3 convertase. Additionally, FH is found in platelet α-granules and is released upon thrombin stimulation or C3b binding to platelets. Notably, FH plays a significant role in downregulating the activation of the classical pathway induced by fibrin clots, suggesting a regulatory function beyond its well-established role in the alternative pathway [56]. Factor H-like protein-1 (FHL-1), derived from an alternatively spliced transcript of the FH gene, retains functional similarity to FH. However, FHL-1 has a lower binding affinity and only possesses one strong C3b-binding site, which limits its regulatory capabilities compared to FH, which has two strong C3b-binding patches [82].

The membrane-bound regulators, CR1 and MCP, serve as cofactors for the factor I-mediated cleavage of C3b and C4b. In addition to its cofactor activity, CR1 exhibits decay-accelerating properties for both C3 and C5 convertases. Similarly, the decay-accelerating factor (DAF), a membrane-bound regulator, primarily functions to accelerate the decay of C3 convertases [77]. The terminal MAC is regulated by the plasma proteins vitronectin and clusterin, and the membrane protein CD59 [46]. Clusterin prevents C9 from binding to the C5b-8 complex, while vitronectin inhibits the formation of the C5b-7 complex as well as C9 polymerisation. As a result, the assembly of the MAC and its subsequent insertion into the membrane is inhibited [81].

Effective complement regulation ensures that activation is localised to pathogens while protecting host cells from collateral damage. Dysregulated complement activity can result in excessive inflammation that damages host tissues, facilitating persistent *Candida* infections. Understanding complement regulation is crucial in the context of *Candida* infections because *Candida* species have been shown to exploit host complement regulators by recruiting them to their cell surface, thereby protecting themselves from complement-mediated destruction.

### 4.2. Recruitment of Human Complement Regulators by Candida

Along with masking and inhibiting complement proteins, *Candida* species can recruit complement regulatory proteins to their surface. These regulatory proteins retain their function, allowing the pathogen to evade complement-mediated attack (Table 3). Several *C. albicans* cell wall-associated proteins including Pra1, Gpm1, high-affinity glucose transporter 1 (Hgt1), and glycerol-3-phosphate dehydrogenase 2 (Gpd2) have demonstrated strong binding affinity to complement regulators such as FH, FHL-1, C4BP, vitronectin, and plasminogen [83]. Interestingly, non-albicans *Candida*, including *C. krusei*, *C. glabrata, C. parapsilosis*, and *C. tropicalis*, also recruit FH and FHL-1; however, the specific fungal proteins mediating these interactions are not well characterised [70].

Pra1, besides functioning as a protease that cleaves C3, is also a surface protein that acquires human proteins, effectively masking the fungal surface. In *C. albicans*, Pra1 is released by both yeast and hyphal forms, with its expression significantly upregulated during the *C. albicans* transition from the yeast to hyphal form, potentially contributing to the heightened invasiveness of the hyphal form [84]. Pra1 binds FH, FHL-1, and plasminogen, each via distinct sites. FH binds Pra1 at two regions within SCR5–7 and SCR16–20, while FHL-1 binds via SCR5–7. Pra1 is the first identified C4BP-binding protein found in *C. albicans*. C4BP interacts with Pra1 through three complement control protein (CCP) domains: CCP4, CCP7, and CCP8. When bound to C4BP, Pra1 regulates the classical and lectin pathways by controlling C3b and C4b deposition and C4b cleavage, thereby favouring *C. albicans* infection [85]. This evasion mechanism is not unique to *C. albicans*—the Pra1 homologue in *C. tropicalis* has been demonstrated to bind FH and C4BP, facilitating the factor I-mediated proteolytic cleavage of C4b and C3b, respectively, affecting all three pathways of complement [76]. Interestingly, Pra1 also enhances FH-mediated complement inactivation in the fluid phase, creating a protective microenvironment for *C. albicans* [84].

Gpm1, a protein found in both the cytoplasm and cell surface of *C. albicans*, plays a dual role in glycolysis regulation and binding to human plasma proteins. Gpm1 was the first fungal protein identified to bind FH and FHL-1, with binding occurring through specific SCR domains: SCR6, SCR7, SCR19, and SCR20 for FH, and SCR6 and SCR7 for FHL-1. Notably, Gpm1 does not interact with C4BP. Additionally, Gpm1 binds plasminogen, enabling its activation to plasmin, which is capable of degrading extracellular matrix components such as vitronectin, hence facilitating the *C. albicans* invasion and colonisation of host tissues [86].

The *C. albicans HGT1* gene encodes a glucose transporter involved in glucose metabolism. Hgt1 is localised to the cell membrane and cell wall, where it plays a dominant role in acquiring FH and C4BP, which disrupt the alternative, classical, and lectin pathway, respectively. Consequently, complement activation on the pathogen surface is downregulated, leading to the reduced formation of MAC and evasion of host defences [87,88].

Gpd2, initially characterised as a cytoplasmic enzyme for glycerol biosynthesis, has also been identified on the surface of both yeast and hyphal forms of *C. albicans*. Gpd2 binds FH and FHL-1 through SCR7, with both regulators retaining their functional activity upon binding. Furthermore, Gpd2 interacts with plasminogen, enabling its conversion to plasmin, which subsequently contributes to complement inhibition. FH, FHL-1, and plasminogen may bind simultaneously to Gpd2 through distinct sites, although they may share some contact points. Notably, the SCR7 domain of FH and FHL-1 in some individuals may harbour a tyrosine-to-histidine polymorphism at position 402 (Y402H), which may influence the binding affinity of Gpd2 and result in increased complement activation [89]. Interestingly, under conditions of elevated oestrogen levels, such as during pregnancy or oral contraceptive use, *C. albicans* adapts by upregulating Gpd2 expression. This upregulation enables the enhanced recruitment of complement regulators, ultimately leading to the reduced activation of the alternative pathway of complement [90].

It has been demonstrated that glucose availability differentially regulates Hgt1 and Gpd2 expression: high glucose levels suppress Hgt1 but upregulate Gpd2, thereby increasing FH deposition on the fungal surface [91]. As a result, complement activation is likely reduced, and the ability of *Candida* species to evade complement-mediated clearance is enhanced. This glucose-dependent regulation might explain why *C. albicans* infections are exacerbated in patients with diabetes, and understanding the metabolic regulation of these complement evasion proteins can inform therapeutic strategies.

The acquisition of soluble human vitronectin onto the fungal surface is another evasion strategy employed by *Candida* species. The surface-associated protein, Gpm1, can bind vitronectin via its C-terminal region, mediating adhesion and enhancing tissue invasion. Vitronectin inhibits C5b-7 complex formation, C9 polymerisation, and its subsequent membrane insertion. As a result, MAC formation is inhibited, providing protection against MAC-mediated lysis [92,93]. Several cell wall- and cytoplasm-derived surface-exposed proteins from *C. tropicalis* and *C. parapsilosis* were found to bind vitronectin [94]. However, whether these interactions confer similar complement evasion properties remains to be elucidated.

Plasminogen appears to be a double-edged sword in complement regulation: while it facilitates complement activation through the proteolytic cleavage of C3 and C5, certain *Candida* species like *C. albicans* and *C. parapsilosis* have evolved mechanisms to exploit its complement inhibitory properties. As previously mentioned, several fungal proteins, including Gpm1, Gpd2, and Pra1, can recruit plasminogen onto the fungal surface, where it is subsequently converted to active plasmin by the urokinase-type plasminogen activator. Plasminogen interacts with complement components C3, C3b, C3d, and C5, enhancing the factor I-mediated cleavage of C3b and thereby promoting complement inactivation. Similarly, plasmin cleaves C3b and C5, further contributing to complement regulation [61]. Notably, there is a difference in the number of plasminogen-binding proteins identified across *Candida* species: eight plasminogen-binding proteins have been identified in *C. albicans* in contrast to the four that have been identified in *C. parapsilosis* [95,96].

The diverse complement evasion strategies employed by different *Candida* species contribute to their differential pathogenicity and host adaptation mechanisms. These strategies may play a significant role in determining the varying levels of virulence observed among different *Candida* species.

**Table 3:** Recruitment of complement regulators as an evasion mechanism employed by different *Candida* species.

Complement Evasion Strategy	Mechanism	Species	References
Recruitment of complement regulators	Pra1 binds FH and FHL-1, resulting in FH-mediated complement inactivation	*C. albicans* and *C. tropicalis*	[76,84,85]
	Pra1 binds C4BP, controlling C3b and C4b deposition and C4b cleavage	*C. albicans* and *C. tropicalis*	[76,85]
	Gpm1 binds FH and FHL-1, resulting in FH-mediated complement inactivation	*C. albicans*	[86]
	Hgt1 acquires FH and C4BP, disrupting AP, CP, and LP	*C. albicans*	[87,88]
	Gpd2 binds FH and FHL-1, resulting in FH-mediated complement inactivation	*C. albicans*	[89]
	Gpm1 binds vitronectin, inhibiting C5b-7 complex formation and C9 polymerisation, providing protection against MAC-mediated lysis	*C. albicans* *C. tropicalis* and *C. parapsilosis*	[92,93,94]
	Gpm1, Gpd2, and Pra1 recruit plasminogen, enhancing FI-mediated cleavage of C3b - Plasminogen cleaves C3b and C5 when activated to plasmin	*C. albicans* and *C. parapsilosis*	[61,85,86,89,95,96]

## 5. Double Trouble: When Drug Resistance Meets Immune Evasion

The emergence of antifungal resistance among *Candida* species represents a significant threat to public health. This, coupled with the immune evasion strategies, including complement evasion, employed by *Candida* species, amplifies the challenge of treating infections. Echinocandins represent the primary therapeutic option for the management of invasive candidiasis, while fluconazole, an azole, serves as an effective alternative first-line treatment for infections caused by susceptible strains due to its high efficiency and good tolerability [97].

Azoles, the most commonly prescribed antifungals for both the treatment and prophylaxis of *Candida* infections, target the enzyme lanosterol 14α-demethylase, a crucial component in the biosynthesis of ergosterol, a sterol component of the fungal cell membrane and mitochondria. By inhibiting this enzyme, azoles reduce cellular ergosterol levels, subsequently leading to the accumulation of toxic sterols and the generation of reactive oxygen species. This fungistatic effect impedes fungal growth without necessarily causing cell death. In contrast, polyenes exhibit fungicidal activity by directly targeting ergosterol, a major sterol component of fungal cell membranes. These agents bind to ergosterol, forming pores that disrupt membrane integrity, resulting in ion leakage and subsequent fungal cell death. Amphotericin B, a prominent polyene, is often reserved for cases where other antifungal classes have proven ineffective. Echinocandins represent another important class of antifungals that exploit the absence of cell walls in mammalian cells. These drugs target the 1,3-β-glucan synthase enzyme, which is essential for fungal cell wall biosynthesis. Echinocandins typically exhibit fungicidal activity and are favoured for their low toxicity profile in humans [98].

Notably, *Candida* species exhibit varying degrees of susceptibility to commonly used antifungals. For instance, *C. albicans* exhibits a fluconazole susceptibility rate of 64.7%, while non-albicans *Candida* species show a lower susceptibility rate of 53.6%. Furthermore, there is an emerging trend of increasing fluconazole resistance among non-albicans *Candida*, with *C. glabrata* displaying significant resistance to azole antifungals [99]. Echinocandins, however, demonstrate potent activity against most *Candida* species, with the exception of *C. parapsilosis* and *C. glabrata* [100,101]. Of particular concern is *C. auris*, the only species with isolates showing resistance to all three major antifungal classes. Notably, caspofungin, an echinocandin typically effective against the biofilms of other *Candida* species, shows limited efficacy against *C. auris* biofilms [102]. While polyene resistance in *Candida* species is relatively rare, which may be attributed to the effectiveness of these drugs in eliminating infections and preventing the evolution of resistant mutants, certain species like *C. auris* exhibit higher rates of resistance to amphotericin B [103].

*Candida* species can acquire antifungal resistance through various mechanisms. These include the overexpression of drug efflux pumps, which has been shown to contribute to azole resistance in *C. glabrata*, *C. parapsilosis*, and *C. auris* [98,104]. This upregulation of efflux transporters effectively reduces intracellular drug concentrations, thereby diminishing antifungal efficacy. Additionally, alteration in the ergosterol biosynthesis pathway, such as the increased expression of lanosterol 14α-demethylase, has been correlated with azole resistance in *C. albicans* and C. *auris* [98]. Furthermore, mutations in genes encoding the 1,3-β-glucan synthase enzyme confer resistance to echinocandins through reduced drug–target binding affinity. Mutations in these genes have been associated with the reduced susceptibility of both *C. parapsilosis* and *C. glabrata* to echinocandins [101,105].

The potential interplay between antifungal resistance development and immune evasion mechanisms in *Candida* species remains to be fully elucidated, although emerging evidence suggests that adaptations serving one purpose simultaneously contribute to the other. Biofilm formation exhibits this dual function: while the extracellular matrix (ECM) acts as a physical barrier with upregulated efflux pumps to expel antifungal agents, it simultaneously masks pathogen-associated molecular patterns (PAMPs) from immune recognition and impairs neutrophil extracellular trap (NET) formation, which are essential for trapping and killing fungi [106].

Similarly, *C. glabrata* and *C. parapsilosis* utilise intracellular niches within macrophages as a survival strategy, wherein prolonged intracellular residence not only enables them to evade immune surveillance and subvert normal phagosome maturation but also may facilitate the development of antifungal resistance through persistent exposure to ineffective drug concentrations [107].

**Figure 2:**
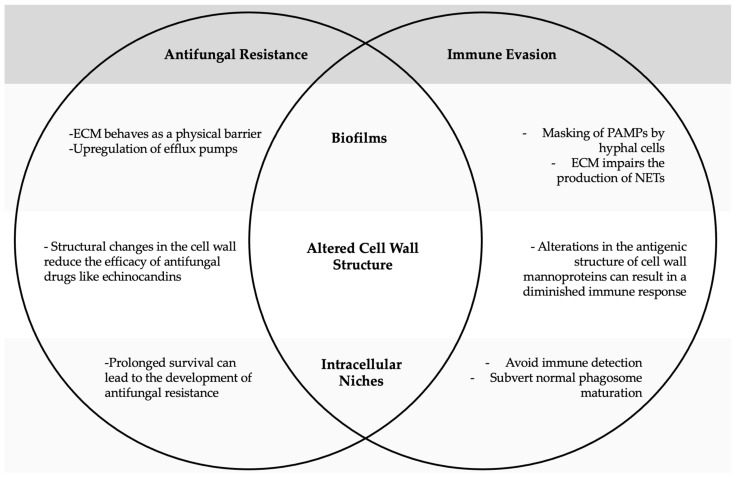
Crosstalk between antifungal resistance and immune evasion. This diagram illustrates how adaptations that enable *Candida* to evade host immune responses can simultaneously contribute to increased antifungal resistance, and vice versa.

Furthermore, it has been reported that *C. glabrata* strains resistant to itraconazole (an azole) and micafungin (an echinocandin) display reduced β-1,2-linked mannose residues. This altered cell wall structure may confer not only resistance to antifungal drugs like echinocandins but also a mechanism to evade host immune responses by downregulating the production of TNF-α, a proinflammatory cytokine [107]. Moreover, the reduction in β-1,2-linked mannose residues may potentially decrease the binding of anti-mannan antibodies and MBL, thus impairing the activation of the lectin pathway. Figure 2 summarises the crosstalk between antifungal resistance and immune evasion.

This emerging antifungal drug resistance represents a significant factor contributing to therapeutic failure in the management of *Candida* infections. The interconnection between immune evasion mechanisms and antifungal resistance frequently results in persistent infections that are increasingly difficult to treat, often necessitating prolonged hospital stays and multiple rounds of antifungal therapy. This not only substantially increases healthcare costs but also places a considerable burden on both patients and healthcare systems. Moreover, the limited effectiveness of current therapeutic approaches emphasises the need for therapeutic strategies that address both aspects simultaneously. Furthermore, there is a pressing requirement for enhanced prevention strategies, including better surveillance systems and improved diagnostic methods for the early detection of resistant strains.

## 6. Conclusions and Future Outlook

Candidiasis represents a significant global healthcare challenge, with its burden further amplified by recent events such as the COVID-19 pandemic. The complement system serves as a critical first-line defence mechanism against invasive *Candida* infections. However, through evolutionary processes, *Candida* species have developed sophisticated mechanisms to evade complement-mediated immune responses. The complexity of these evasion strategies, involving multiple proteins, suggests that single-target therapeutic approaches may be inadequate for effective treatment.

While *C. albicans* remains the most extensively studied species, the increasing prevalence of non-albicans *Candida* species, coupled with their diverse antifungal susceptibilities, underscores the importance of extending research across different *Candida* species. Future research should prioritise molecular and cellular studies of complement and other immune evasion strategies in non-albicans *Candida* species, focusing on potential novel proteases capable of cleaving complement proteins and unidentified surface proteins that may recruit complement regulators.

The emergence of drug-resistant *Candida* species like *C. auris* necessitates the development of novel antifungal therapeutic strategies. Several promising candidates are currently advancing through phase II and phase III clinical trials, including rezafungin, ibrexafungerp, and fosmanogepix, which have advantages like a prolonged plasma half-life or broader antifungal properties [108]. While anti-*Candida* vaccine development represents another promising therapeutic avenue, significant challenges persist, such as the target population being immunocompromised, raising concerns about the safety and efficacy of the potential vaccine in these vulnerable populations. Additionally, the considerable phenotypic and virulence variation, both within and between *Candida* species, presents challenges for developing a universally effective vaccine.

In conclusion, understanding the molecular mechanisms underlying complement evasion can inform the identification of specific therapeutic targets. Furthermore, elucidating the crosstalk between antifungal resistance and immune evasion mechanisms may facilitate the development of immunotherapeutic approaches which could potentially combat resistance mechanisms while simultaneously restoring the efficacy of existing antifungal agents, ultimately leading to more effective treatment options for invasive candidiasis.

## Data Availability

This study did not generate any new data. All data analysed in this review are publicly available in the cited research studies and can be accessed through their relevant publications in the reference list.
